# Retraction: Hsa-miR-139-5p inhibits proliferation and causes apoptosis associated with down-regulation of c-Met

**DOI:** 10.18632/oncotarget.28329

**Published:** 2023-01-26

**Authors:** Chengcao Sun, Ming Sang, Shujun Li, Xiaodong Sun, Cuili Yang, Yongyong Xi, Liang Wang, Feng Zhang, Yongyi Bi, Yunfeng Fu, Dejia Li

**Affiliations:** ^1^Department of Occupational and Environmental Health, School of Public Health, Wuhan University, 430071 Wuhan, P.R. China; ^2^Central Laboratory of the Fourth Affiliated Hospital in Xiangyang, College of Basic Medical Sciences, Hubei Key Laboratory of Wudang Local Chinese Medicine Research, Hubei University of Medicine, 442000 Shiyan, P.R. China; ^3^Institute of Global Health, Wuhan University, 430071 Wuhan, P.R. China; ^4^Wuhan Hospital for the Prevention and Treatment of Occupational Diseases, 430071 Wuhan, P.R. China; ^5^The Third Xiang-ya Hospital of Central South University, 410013 Changsha, P.R. China; ^*^These authors have contributed equally to this work

**This article has been retracted:** Oncotarget requested the original data for this paper to address concerns regarding image duplication. The authors were unable to retrieve the data. As a result, all authors have agreed to the retraction of this paper from Oncotarget.

Original article: Oncotarget. 2015; 6:39756–39792. 39756-39792. https://doi.org/10.18632/oncotarget.5476

